# Bioresorbable scaffolds vs. drug-eluting stents for patients with myocardial infarction: A systematic review and meta-analysis of randomized clinical trials

**DOI:** 10.3389/fcvm.2022.974957

**Published:** 2022-10-28

**Authors:** Yong Liu, Di Xiao, Yang Wu, Meng Li, Jia Liu, Rui Zhuang, Liyong Ma, Jingen Li, Lijing Zhang

**Affiliations:** ^1^Department of Cardiology, Dongfang Hospital, Beijing University of Chinese Medicine, Beijing, China; ^2^Department of Cardiology, Dongzhimen Hospital, Beijing University of Chinese Medicine, Beijing, China

**Keywords:** bioresorbable scaffold, drug-eluting stents, myocardial infarction, meta-analysis, randomized controlled trial

## Abstract

**Objective:**

To compare the efficacy and safety of bioresorbable scaffolds (BRS) with drug-eluting stents (DES) in patients with myocardial infarction undergoing percutaneous coronary interventions (PCI).

**Methods:**

We performed a systematic review and meta-analysis of randomized controlled trials (RCTs) comparing BRS with DES on clinical outcomes with at least 12 months follow-up. Electronic databases of PubMed, CENTRAL, EMBASE, and Web of Science from inception to 1 March 2022 were systematically searched to identify relevant studies. The primary outcome of this study was the device-oriented composite endpoint (DOCE) consisting of cardiac death, target-vessel myocardial infarction, and target lesion revascularization. Secondary outcomes were a composite of major adverse cardiac events (MACE, all-cause death, target-vessel myocardial infarction, or target vessel revascularization) and the patient-oriented composite endpoint (POCE, defined as a composite of all-cause death, myocardial infarction, or revascularization). The safety outcomes were definite/probable device thrombosis and adverse events.

**Results:**

Four randomized clinical trials including 803 participants with a mean age of 60.5 ± 10.8 years were included in this analysis. Patients treated with BRS had a higher risk of the DOCE (RR 1.62, 95% CI: 1.02–2.57, *P* = 0.04) and MACE (RR 1.77, 95% CI: 1.02–3.08, *P* = 0.04) compared with patients treated with DES. No significant difference on the POCE (RR 1.33, 95% CI: 0.89–1.98, *P* = 0.16) and the definite/probable device thrombosis (RR 1.31, 95% CI: 0.46–3.77, *P* = 0.61) were observed between BRS and DES. No treatment-related serious adverse events were reported.

**Conclusion:**

BRS was associated with a higher risk of DOCE and MACE compared with DES in patients undergoing PCI for myocardial infarction. Although this seems less effective in preventing DOCE, BRS appears as safe as DES.

**Systematic review registration:**

[https://www.crd.york.ac.uk/PROSPERO/display_record.php?RecordID=321501], identifier [CRD 42022321501].

## Introduction

The vascular stent has been considered one of the landmark advancements in interventional cardiology. Even though the new generation of drug-eluting stents (DES) using stainless steel with a polymer coating carrying anti-cell-proliferative drugs has greatly reduced the risk of stent thrombosis (ST), target lesion revascularization (TLR), and other major adverse cardiac events (MACE) (1–4), in-stent restenosis caused by neointimal hyperplasia or ST induced by suppression of endothelial cells are often observed in DES (5). There is a 2–3% annual incidence of device-related adverse events (AEs) 1 year after stent implantation regardless of stent type (6). This hazard has been attributed to the presence of a metallic implant that distorts and constrains the vessel, causing chronic inflammation and vascular remodeling, finally leading to very late events consisting of target vessel revascularization, target-vessel myocardial infarction, and TLR (7, 8). In addition, the DES has been found to cause remarkable alteration in the electrical parameters of the erythrocyte membrane, indicating that the full biocompatibility of current metal stents has not yet been reached (9). The bioresorbable scaffolds (BRS) which provide similar mechanical support and drug release with DES early after implantation but could be completely absorbed later were thus developed to overcome problems associated with metallic stents remaining in the coronary arteries for long periods (10, 11). And the physiological advantages of BRS, such as late lumen enlargement and vasomotion, are particularly appealing for coronary revascularization. However, evidence on the safety and efficacy of BRS vs. DES in patients with myocardial infarction is inconsistent; some trials (12–14) showed that BRS was similar to or better than DES on a device-oriented endpoint, while other trials reported that BRS was associated with higher risk TLR (15, 16). Therefore, we conducted this systematic review and meta-analysis to compare the efficacy and safety of BRS vs. DES in patients with myocardial infarction undergoing percutaneous coronary interventions (PCI).

## Methods

Our review was registered at PROSPERO (CRD 42022321501) and was performed according to the PRISMA guidelines (17) and the Cochrane Collaboration recommendations (18).

### Study eligibility criteria

Trials were included in the analysis if they fit the following criteria: (1) RCTs, only randomized controlled trials were included to avoid confounding; (2) participants with established myocardial infarction, including ST-segment elevation myocardial infarction and non-ST-segment elevation myocardial infarction; (3) comparing BRS with DES; (4) with a follow-up of at least 12 months; and (5) reporting at least one of the study outcomes.

### Study outcomes

The device-oriented composite endpoint (DOCE), consisting of cardiac death, target-vessel myocardial infarction, and TLR was the main outcome measure for our study. MACE (defined as a composite of all-cause death, target-vessel myocardial infarction, or target vessel revascularization) and the patient-oriented composite endpoint (POCE, all-cause death, myocardial infarction, or revascularization) were the secondary outcomes. The major safety outcome measures were definite/probable device thrombosis and AEs. We abstracted the outcome data at the end of study follow-up and used the longest follow-up reported for each study.

### Search methods

In accordance with PRISMA guidelines (17), we searched PubMed, CENTRAL, EMBASE, and Web of Science with the keywords “everolimus-eluting stent,” “drug-eluting stent,” “Xience,” “BVS,” “BRS,” “bioresorbable scaffold,” “bioabsorbable scaffold,” “bioabsorbable stent,” “bioresorbable stent,” and “randomized trial” up to 1 March 2022. No restrictions were applied concerning language. The detailed search strategies, which were constructed using “BRS,” “DES,” and “myocardial infarction” for PubMed, are presented in Supplementary material.

### Data collection and analyses

Two authors (YL and DX) performed study screening and data extraction independently, with disagreement resolved by discussion and consensus, and a third author was consulted when no consensus was reached. The following data were extracted: study characteristics (e.g., study date and location), participants (e.g., age and gender), control (i.e., DES and everolimus-eluting stents), interventions (i.e., BRS and bioresorbable everolimus-eluting vascular scaffolds), and outcomes (e.g., cardiac death, target-vessel myocardial infarction, and TLR). When reported data were insufficient for analysis, we contacted the study authors for additional data.

### Risk of bias assessment

Each study was assessed independently the risk of bias by two authors (RZ and LM) using the Cochrane risk of bias tool (18), evaluating selection bias, performance bias, detection bias, attrition bias, reporting bias, and other biases of the included trials.

### Publication bias risk

Publication bias was planned to be evaluated by funnel plot inspection when there were 10 or more trials. However, due to the insufficient RCTs included in our study, risk of publication bias was not performed.

### Data synthesis

Categorical variables are reported as frequencies with percentages, and we reported continuous variables as mean ± SD or median (interquartile range). The dichotomous pooled outcomes were calculated as risk ratios (RRs) with 95% confidence interval (CI) and we calculated mean differences (MDs) or standardized mean differences (SMDs) with 95% CI for continuous data. As a measure of between-study heterogeneity, *I*^2^ was calculated; *I*^2^ values of 25, 50, and 75% were interpreted as mild, moderate, and severe between-study heterogeneity, respectively. A fixed-effects model was used when study-specific risk estimates were homogeneous; otherwise, a random-effects model was used. The funnel plots was used to assess publication bias. The Stata software (version 17.0; Stata Corporation, College Station, TX, USA) and REVMAN software (version 5.4; Cochrane Collaboration, 2020) were used to verify and analyze the clinical data.

### Subgroup analysis

Subgroup analyses, based on different populations (e.g., ST-segment elevation myocardial infarction vs. non-ST-segment elevation myocardial infarction) and the comprehensiveness of each intervention, were planned. Nevertheless, due to insufficient data, we were unable to perform the preplanned analyses.

## Results

### Literature search

**FIGURE 1:**
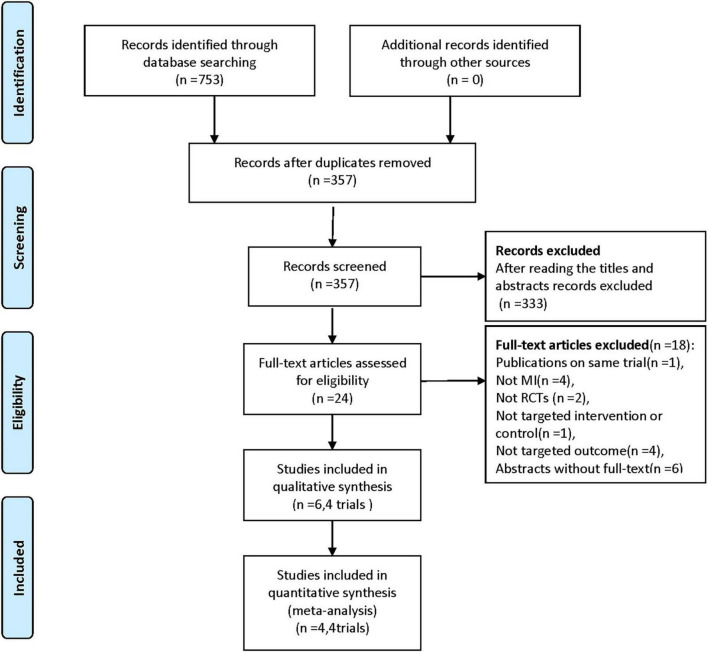
Flowchart for the trial selection. RCTs, randomized controlled trials; MI, myocardial infarction.

The literature search and screening process are shown in Figure 1. After the initial search of electronic databases, a total of 753 citations were retrieved. And 24 full-text articles were reviewed for eligibility after removing duplications and screening titles and abstracts. Ultimately, 4 RCTs [6 records (15, 19–23)] with a total of 803 participants treated with BRS or DES were included (15, 19, 21, 23) (excluded records were listed in Supplementary material).

### Characteristics of included studies and participants

Patients were randomized to be treated with Absorb™ everolimus-eluting BRS (Abbott Vascular, Santa Clara, CA, USA/Magmaris^®^, Biotronik Ag, Bülack, Switzerland), bioresorbable everolimus-eluting scaffolds BVS, the XIENCE metallic everolimus-eluting stents (Abbott Vascular), or sirolimus-eluting stents (Orsiro^®^, Biotronik AG, Bülack, Switzerland). Baseline patient characteristics are shown in Tables 1, 2. And 81.9% of patients included in this analysis were male with a mean age of 60.5 ± 10.8 years, 51.0% had hypertension, 55.1% had dyslipidemia, 18.4% had diabetes mellitus, 9.0% had a preceding history of myocardial infarction, 10.2% had a prior history of PCI, and 41.9% were smokers. The prevalence of the other comorbidities, that is, obesity and chronic obstructive pulmonary disease (COPD) were only reported in two trials, which demonstrate no significant between-group difference (15, 21, 23). Three trials (15, 21, 23) reported antiplatelet/anticoagulant therapy during the procedure and the difference between groups was not statistically significant. In addition, two trials reporting dual antiplatelet therapy at 1-year follow-up showed no differences between BRS and DES groups in dual antiplatelet use. Lesion and procedural characteristics are shown in Table 3. The culprit vessels were present in the left anterior descending artery in 409 patients (51.6%), in the right coronary artery in 313 patients (39.5%), and the left circumflex artery in 138 patients (17.4%). All angiograms were mainly analyzed by quantitative coronary angiography (QCA) in the four included trials, except in the case of one trial (19). Regarding lesion preparation, pre-dilatation was performed in 80.3% and post-dilatation was performed in 53.4% of all included participants. Follow-up data on clinical outcomes were available in 98.6% (*n* = 792) of all included patients [98.0% (435/444) BRS vs. 99.4% (357/359) DES].

**TABLE 1:** Clinical characteristics of included trials.

References	Region	Target population	Sample size (I/C)	Mean age (I/C)	Male (I/C))	Intervention	Control	Follow-up (months)	AEs	DAPT (I/C)
de la Torre Hernandez et al. (19)	Spain	MI	100/100	60.8 ± 11.0/61.3 ± 12.0	79/76	BVS	EES	12	NA	NA
Sabaté et al. (15)	Spain	STEMI	74/76	58.8 ± 10.6/59.2 ± 10.3	63/71	BRS	SES	12	I:1 C:2	I: 63 (90.0) C: 62 (89.9)
Wiebe et al. (23)	Germany	STEMI/NSTEMI	173/89	61.7 ± 11.0/63.3 ± 9.9	138/65	BRS	EES	24	NA	NA
Katagiri et al. (21)	Multicenter	STEMI	95/96	59.1 ± 10.7/58.2 ± 9.6	73/84	BVS	EES	36	NA	I: 73 (78.5) C: 75 (78.1)

MI, myocardial infarction; STEMI, ST-segment elevation myocardial infarction; NSTEMI, non-ST-segment elevation myocardial infarction; BVS, bioresorbable everolimus-eluting vascular scaffolds; BRS, bioresorbable scaffolds; EES, everolimus-eluting stents; SES, sirolimus-eluting stent; I/C, intervention/control; Multicenter, Denmark, Netherlands, Spain, Switzerland; AEs, adverse events; NA, not applicable; DAPT, dual antiplatelet therapy (dual antiplatelet therapy at 1-year follow-up).

**TABLE 2:** Clinical characteristics of included trials.

	Wiebe et al. (23)		Sabaté et al. (15)		Katagiri et al. (21)		de la Torre Hernandez et al. (19)	
				
	BRS	EES	BRS	SES	BVS	EES	BVS	EES
BMI, kg/m^2^	27.0 ± 4.0	27.0 ± 3.7	NA	NA	27.0 ± 4.1	27.7 ± 4.2	NA	NA
Diabetes, *n* (%)	37 (21.6)	17 (19.3)	10 (13.5)	14 (18.4)	18 (18.9)	14 (14.7)	16 (16.0)	20 (20.0)
Dyslipidemia, *n* (%)	74 (43.5)	40 (47.6)	50 (67.6)	37 (48.7)	60 (63.8)	55 (57.3)	58 (58.0)	62 (62.0)
Hypertension, *n* (%)	92 (53.5)	54 (62.1)	33 (44.6)	32 (42.1)	41 (44.1)	35 (36.5)	56 (56.0)	61 (61.0)
Smoking, *n* (%)	77 (44.5)	38 (43.2)	41 (55.4)	43 (56.6)	46 (48.4)	47 (49.5)	21 (21.0)	19 (19.0)
COPD, *n* (%)	NA	NA	2 (2.7)	6 (7.9)	3 (3.2)	3 (3.1)	NA	NA
Prior MI, *n* (%)	12 (6.9)	6 (6.7)	5 (6.8)	3 (3.9)	2 (2.1)	3 (3.1)	18 (18.0)	22 (22.0)
Prior PCI, *n* (%)	15 (8.8)	7 (7.9)	3 (4.1)	2 (2.6)	4 (4.2)	3 (3.1)	21 (21.0)	26 (26.0)

CAD, coronary artery disease; MI, myocardial infarction; PCI, percutaneous coronary intervention; BVS, bioresorbable everolimus-eluting vascular scaffolds; BRS, bioresorbable scaffolds; EES, everolimus-eluting stents; SES, sirolimus-eluting stent; NA, not applicable; COPD, chronic obstructive pulmonary disease; BMI, body mass index.

**TABLE 3:** Lesion and procedural characteristics of included trials.

	Wiebe et al. (23)		Sabaté et al. (15)		Katagiri et al. (21)		de la Torre Hernandez et al. (19)	
				
	BRS	EES	BRS	SES	BVS	EES	BVS	EES
**Target vessel**								
LAD, *n* (%)	82 (47.4)	43 (48.3)	36 (48.6)	36 (47.4)	34 (35.8)	41 (41.8)	66 (52.8)	71 (54.6)
LCX, *n* (%)	30 (17.3)	10 (11.2)	16 (21.6)	11 (14.5)	17 (17.9)	13 (13.3)	19 (15)	22 (16.9)
RCA, *n* (%)	61 (35.3)	36 (40.4)	22 (29.7)	29 (38.1)	44 (46.3)	44 (44.9)	40 (32)	37 (28.4)
Stent diameter (mm)	3.2 ± 0.4	3.2 ± 0.4	3.5 ± 0.2	3.3 ± 0.4	3.2 ± 0.3	3.12 ± 0.37	3.08 ± 0.4	3.01 ± 0.5
Pre-dilatation, *n* (%)	164 (95.3)	72 (81.8)	72 (91.1)	70 (86.4)	53 (55.8)	50 (51.0)	122 (97.6)	33 (25.4)
Post-dilation, *n* (%)	98 (56.6)	31 (34.8)	70 (88.6)	20 (24.7)	48 (50.5)	25 (25.5)	81 (64.8)	50 (38.4)
Device success, *n* (%)	NA	NA	73 (98.6)	76 (100.0)	91 (95.8)	98 (100.0)	NA	NA
**Medication during procedure**								
Heparin, *n* (%)	167 (96.5)	85 (97.7)	69 (93.2)	74 (97.4)	86 (90.5)	85 (86.7)	NA	NA
Bivalirudin, *n* (%)	1 (0.6)	1 (1.1)	1 (1.4)	0 (0.0)	25 (26.3)	22 (22.4)	NA	NA
GP IIb/IIIa antagonists, *n* (%)	0 (0)	0 (0)	14 (18.9)	15 (19.7)	38 (40.0)	37 (37.8)	NA	NA
Imaging assessment tools	QCA		QCA		QCA		QCA/OCT/IVUS	

LAD, left anterior descending; LCX, left circumflex; RCA, right coronary artery; BVS, bioresorbable everolimus-eluting vascular scaffolds; BRS, bioresorbable scaffolds; EES, everolimus-eluting stents; SES, sirolimus-eluting stent; NA, not applicable; QCA, Quantitative coronary angiography; OCT, Optical coherence tomography; IVUS, Intravascular ultrasound; Device success, defined as the implantation of the assigned study device with a post-procedure residual stenosis <30%.

### Risk of bias

Detailed bias assessments are provided in Supplementary Table 1. The Random sequence generation was described in detail in all included trials (15, 19, 21, 23). Therefore, all included trials were at low risk for selection bias. Regarding sequence generation and allocation concealment, only three trials (15, 21, 23) reported that the allocation schedule was generated by computer, while the remaining one trial (19) did not report how the sequence allocation was conducted and was thus assessed as high risk of selection bias. All four included trial were assessed as high risk of performance bias due to the open-label design. Concerning the blinding of outcome assessment, it was graded as low since the outcomes of interest were clinical events (i.e., myocardial infarction, cardiac death, etc.) which are objectives and well-defined endpoints. The attrition bias also was low given that 11 patients were lost to follow-up for all-cause death in one trial (23) [5.2% (9/173) BRS vs. 2.2% (2/89) DES] and three trials (15, 19, 21) reported no patient was lost to follow-up. Regarding the selective reporting of outcomes, one trial (19) was considered to have an unclear risk due to the lack of study protocol.

### Treatment outcomes

#### Device-oriented composite endpoint

**FIGURE 2:**
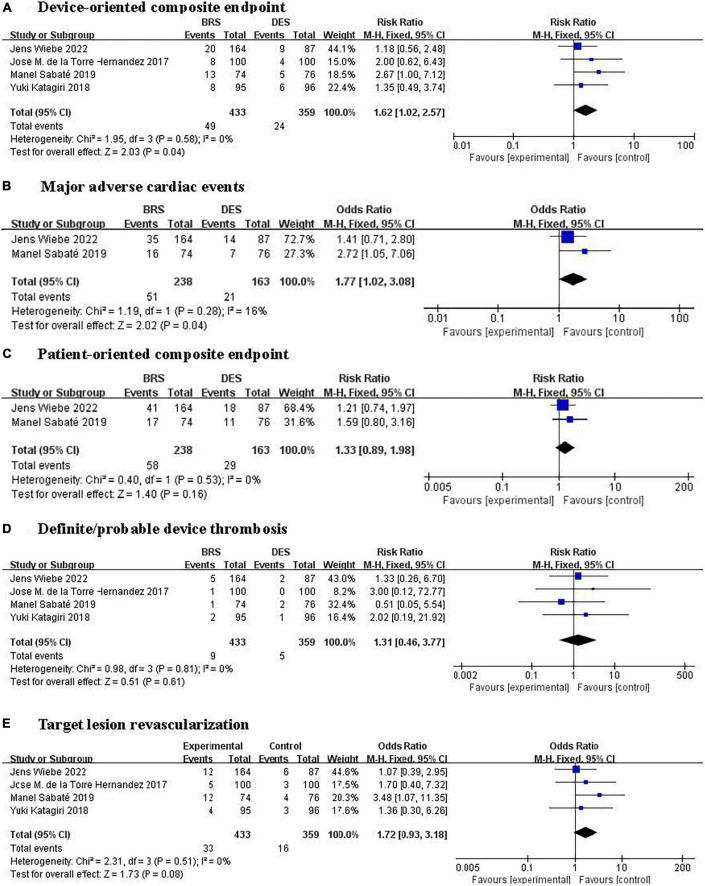
Forest plots of effect of BRS and DES on clinical outcomes. **(A)** Device-oriented composite endpoint, **(B)** major adverse cardiac events, **(C)** patient-oriented composite endpoint, **(D)** definite/probable device thrombosis, and **(E)** target lesion revascularization. BRS, bioresorbable scaffolds; DES, drug-eluting stents.

A total of four trials (15, 19, 21, 23) including 792 patients reported the primary outcome of DOCE and it had occurred in 73 patients [11.3% (49/433) BRS vs. 6.7% (24/359) DES]. Pooled results showed that compared with DES-treated patients, patients treated with BRS had a higher risk of DOCE (RR 1.62, 95% CI: 1.02–2.57, *P* = 0.04; *I*^2^ = 0%; Figure 2A).

#### Major adverse cardiac events

Two trials (15, 23) involving 401 participants reported the composite outcome of MACE consisting of all-cause death, target-vessel myocardial infarction, and target vessel revascularization occurred in 72 patients [21.4% (51/238) BRS vs. 12.9% (21/163) DES]. Pooled results showed that compared with DES-treated patients, participants treated with BRS showed a higher risk of MACE (RR 1.77, 95% CI: 1.02–3.08, *P* = 0.04, *I*^2^ = 16%; Figure 2B). One trial (21) reported results separately for target-vessel myocardial infarction and all-cause death events, while another (19) reported only target-vessel myocardial infarction. And the risk of target-vessel myocardial infarction or all-cause death between BRS and DES had no significant differences.

#### Patient-oriented composite endpoint

The POCE was reported in four trials (15, 19, 21, 23) (792 participants) with two trials (15, 23) reporting the POCE (defined as a composite of all-cause death, myocardial infarction, or revascularization), one trial (21) reporting all-cause death and myocardial infarction, and the remaining one trial (19) reporting only myocardial infarction. Two trials (15, 23) involving 401 participants reported the POCE occurred in 87 patients [24.4% (58/238) BRS vs. 17.8% (29/163) DES]. Pooled results of two trials (15, 23) comparing BRS with DES showed a 33% increase in risk of POCE in BRS group; however, this difference does not reach statistical significance (RR 1.33, 95% CI: 0.89–1.98, *P* = 0.16, *I*^2^ = 0%; Figure 2C).

#### Definite/probable device thrombosis

All studies (15, 19, 21, 23) reported the main safety outcome of definite/probable device thrombosis, which had occurred in 14 patients [24.4% (9/433) BRS vs. 17.8% (5/359) DES]. No statistically significant differences were found in the risk of definite/probable device thrombosis between BRS and DES (OR 1.31, 95% CI: 0.46–3.77, *P* = 0.61; *I*^2^ = 0%; Figure 2D).

#### Adverse events

No treatment-related serious AEs were reported in either BRS or DES group, only one trial (15) had reported that the definite device thrombosis occurred in one patient treated with BRS implantation after 20 min, which was addressed by thrombectomy and new balloon post dilatation, while thromboembolic event occurred in two patients treated with DES.

## Discussion

Our systematic review and meta-analysis found that compared with DES, BRS was related to a higher risk of the DOCE (cardiac death, target-vessel myocardial infarction, or TLR) and MACE (all-cause death, target-vessel myocardial infarction, or target vessel revascularization) in patients with myocardial infarction. A trend toward a higher risk of TLR of BRS was also observed (Figure 2E). However, the BRS did not differ from DES on the definite/probable device thrombosis and the POCE (all-cause death, myocardial infarction, or revascularization).

### Comparison with previous studies

A previous meta-analysis conducted in patients with coronary artery disease consisting of myocardial ischemia, coronary artery stenosis, and myocardial infarction showed that BRS was associated with higher risk of the DOCE and stents thrombosis cumulatively at 2 years of follow-up than DES (9). And another meta-analysis conducted in patients with acute coronary syndrome (ACS, including unstable angina, STEMI, and NSTEMI) showed that BRS was linked to a higher risk of TLR at a median follow-up of 9.5 months, which was mainly driven by device thrombosis (24). In the present study, we found, for the first time, that even in patients with myocardial infarction consisting of STEMI and NSTEMI, BRS was associated with a greater risk of device-oriented events.

Consistent with our findings, Collet et al. (25) compared BRS and everolimus-eluting stents on long-term clinical outcomes in patients with obstructive coronary artery disease and found that BRS was related to a higher risk of definite thrombosis and a trend toward higher target lesion failure risk. Ali et al. (7) had done a systematic review of BRS for coronary artery disease in 2017, but many participants with complex lesions and high-risk were excluded. A meta-analysis done in 2018 by De Rosa et al. (24) observed that patients with ACS undergoing PCI with BRS vs. everolimus-eluting stents have a higher risk of TLR at a median follow-up of 9.5 months. However, as the timepoint of the complete bioresorption of BRS in humans is unclear, the median follow-up of 9.5 months may not be insufficient. Notably, none of the above studies focused on patients with myocardial infarction consisting of STEMI and NSTEMI. In the past few years, new evidence has emerged regarding the effects of BRS on myocardial infarction. These most recent studies were included in our study and we provided the first pooled evidence of BRS on clinical outcomes for myocardial infarction.

### Safety of bioresorbable scaffolds

Consistent with previous studies, we observed that the incidence of definite/probable device thrombosis did not differ between the BRS and the DES group. In patients with myocardial infarction undergoing PCI, Byrne et al. (22) found that the rates of device thrombosis [3 (1.7%) vs. 2 (2.3%), HR 0.76, 95% CI 0.13–4.56] were similar in BRS and DES group at 1 year. The MAGSTEMI trial (15), which was powered for clinical events, demonstrated no significant difference between bioresorbable everolimus-eluting vascular scaffolds and everolimus-eluting stents [1 (1.4%) in the BRS arm vs. 2 (2.6%), *P* = 1.0] in the risk of definite device thrombosis at 1-year follow-up. Similar results were obtained by two other independent studies (21, 23), the 2- and 3-year definite/probable device thromboses were 3.0% (23) and 2.1% (18), respectively. Meta-analysis of four studies including 3,384 patients with coronary artery disease (26) revealed that no difference in hazards of bioresorbable everolimus-eluting vascular scaffolds on ST after 3 years of follow-up, but at 3 to 5 years, bioresorbable everolimus-eluting vascular scaffolds showed a lower risk of ST than everolimus-eluting stents. This suggested that BRS may reduce risk of the long-term ST. The BRS-treated patients were at a higher risk of ST, which might be partially explained by its material and composition. First, metal stents have a smooth surface produced by the electropolishing process, which can help prevent the activation and aggregation of platelets in thrombosis processing (27), but polymeric scaffolds cannot undergo the same process. Surface roughness of BRS may activate the process of thermogenesis and stimulate tissue reaction and thus leads to higher risk of ST and in-stent restenosis. Second, thrombus dissolution may lead to stent strut malposition in the first few months for patients with myocardial infarction after PCI due to the high risk of thrombosis with BRS, causing a higher incidence of AEs (27).

### Potential mechanism

We found in the present analysis that patients with myocardial infarction treated with BRS had a higher risk of the DOCE and MACE than patients treated with DES. The underlying reasons for our findings are as the follows: First, the previous study (28) has already illustrated that the risk of structural disruption/fracture is likely to limit the over-expansion possibility of BRS, especially the first-generation device, which may result in a higher risk of AEs in the follow-up. Second, compared with metallic DES, the BRS implantation in vessels with reference diameter <2.25 mm might lead to increased rates of target lesion failure (12.9 vs. 8.3%) and device thrombosis (4.6 vs. 1.5%) (29). Part of the above-mentioned problem can be attributed to their bulky struts, especially in the overlapping scaffolds, which could generate a thickness of 300–400 μm, disturbing the effective flow area in small coronary lumens. Third, patients with myocardial infarction, especially those with STEMI, have thrombus-rich lesions with a large necrotic core, which are usually related to delayed arterial healing and impaired stent-related outcomes. Also, special attention should be paid to the size of blood vessels, as coronary spasm is often observed in the STEMI setting and might lead to scaffold undersize. Fourth, an inflammatory response is necessary to resolve the necrotic myocardium after acute myocardial infarction and vascular inflammation is an essential component of atherosclerosis that result in plaque instability and cardiovascular events (30, 31). Patients with myocardial infarction have a higher risk of additional major adverse cardiovascular events. Previous study showed that myocardial infarction accounts for 46% of all deaths attributed to cardiovascular diseases (32).

### Implication for future study

In our study, only studies with at least 1 year of follow-up were included, and a longer term of follow-up is need as it seems that BRS has greater long-term benefit than DES. In future, prospective RCTs with longer duration and larger samples are needed to confirm these findings. Apart from myocardial infarction, ischemic heart disease (IHD) also represents a large burden on individuals and healthcare resources worldwide. Unlike myocardial infarction, clinical, angiographic, and autoptic findings suggest a multifaceted pathophysiology (e.g., endothelial dysfunction, vasospasm, or coronary microvascular dysfunction) for IHD and atherosclerotic plaques only accounts for a small part of the pathophysiology (33). Therefore, future studies may focus on effects of different types of stents on other aspects of pathophysiology of IHD, such as endothelial dysfunction.

### Study limitations

Some limitations are inherent to our systematic review and meta-analysis that should be noted. First, results of the present analysis should be interpreted with caution due to the small sample size and the potential risk of bias. Second, previous studies have demonstrated that there are some differences between the pathophysiology underlying STEMI and NSTEMI populations, the pathophysiology of the culprit artery is typically non-occlusive thrombotic plaque rupture and subendocardial infarction in NSTEMI (34), and STEMI populations have been found to have an increased pro-inflammatory state and a different serological profile (35). Thus, it has to be admitted that the inclusion of both STEMI and NSTEMI patients introduced heterogeneity into the study population. But due to the small number of available trials, we were unable to conduct subgroup analysis according to STEMI and NSTEMI. However, as limited as it might be, our findings did provide the only available pooled evidence for the comparison of BRS with DES in patients with myocardial infarction. Third, because of a paucity of relevant data in the original literature, we were unable to compare the distribution of potential confounders, such as chronic kidney disease (CKD), COPD, and autoimmune diseases. However, the RCT design may have minimized the effects of these potential confounders. Fourth, secondary prevention medication use (e.g., aspirin or statins) was not properly reported in the original study, which could also lead to a biased conclusion. Finally, although the results of MACE assessed in our study were statically significant, the two included trials are too small to provide strong evidence. Therefore, future studies are required to verify the effects of BRS for MACE.

## Conclusion

Bioresorbable scaffolds implantation was associated with a higher risk of the DOCE and MACE compared with DES in patients with myocardial infarction undergoing PCI. Although seems less effective in preventing DOCE, BRS appears as safe as DES.

## Data availability statement

The original contributions presented in the study are included in the article/Supplementary material, further inquiries can be directed to the corresponding authors.

## Author contributions

YL and DX conceived and designed the protocol, developed and implemented the search strategy, extracted the data, resolved the discrepancies, and wrote the first draft of the manuscript. JGL refined the protocol and submitted it to PROSPERO. ML and JL screened the retrieved records. RZ, LM, and YL were responsible for the risk of bias in all studies. ML, YW, LJZ, and JGL refined the manuscript. All authors have approved the final draft of the manuscript.
