# Supplementary material for: Bioresorbable scaffolds vs. drug-eluting stents for patients with myocardial infarction: A systematic review and meta-analysis of randomized clinical trials
Source: Front Cardiovasc Med. 2022 Oct 28;9:974957. doi: 10.3389/fcvm.2022.974957 (PMC9649707; doi:10.3389/fcvm.2022.974957)
Supplement: Supplementary file 1 [file Presentation_1.pdf]

Supplementary Online Content

Table of contents:

1. Risk of bias assessment (Table 1)…………………………………………………...3

2. The search strategy of Pubmed……………………………………………………...5

3. Excluded records after full-text screening…………………………………………...6

4. REFERENCES……………………………………………………………. ……...9

Table 1. Risk of bias assessments for randomized clinical trials(1).

	Random sequence generation (selection bias)	Allocation concealment (selection bias)	Blinding of participants and researchers (performance bias)	Blinding of outcome assessment (detection bias)	Incomplete outcome data (attrition bias)	Selective reporting (reporting bias)	Other bias	Overall
**Jens Wiebe 2022 (5)**	LOW	LOW	HIGH	LOW	LOW	LOW	LOW	LOW
**Manel Sabaté 2019 (6)**	LOW	LOW	HIGH	LOW	LOW	LOW	LOW	LOW
**Yuki Katagiri 2018 (4)**	LOW	LOW	HIGH	LOW	LOW	LOW	LOW	LOW
**Jose M. de la Torre Hernandez 2017 (3)**	LOW	HIGH	HIGH	LOW	LOW	unclear	LOW	HIGH

Random sequence generation were all described in detail in all included trials (2-5). Therefore, all included trials were assessed as low risk of selection bias. For the allocation sequence concealment, three trials (3-5) reported the allocation schedule was generated by computer, while the remaining one trial (2) did not report how the sequence allocation was conducted and was thus assessed as high risk of selection bias. All four included trial were assessed as high risk of performance bias due to the open-label design. Concerning the blinding of outcome assessment, it was graded as low since the outcomes of interest were clinical events (i.e., myocardial infarction, cardiac death, etc.) which are objectives and well-defined endpoints. The attrition bias also was low given that 11 patients were lost to follow-up for all-cause death in one

trial (4) (5.2% [9/173] BRS vs. 2.2% [2/89] DES) and three trials (2,3,5) reported no patient was lost to follow-up. Regarding the selective reporting of outcomes, one trial (2) was considered to have an unclear risk due to the lack of study protocol.

The search strategy of PubMed:

1. ("Myocardial Infarction"[Mesh])
2. ((((((((((((Infarction, Myocardial[Title/Abstract]) OR (Infarctions, Myocardial[Title/Abstract])) OR (Myocardial Infarctions[Title/Abstract])) OR (Cardiovascular Stroke[Title/Abstract])) OR (Cardiovascular Strokes[Title/Abstract])) OR (Stroke, Cardiovascular[Title/Abstract])) OR (Strokes, Cardiovascular[Title/Abstract])) OR (Myocardial Infarct[Title/Abstract])) OR (Infarct, Myocardial[Title/Abstract])) OR (Infarcts, Myocardial[Title/Abstract])) OR (Myocardial Infarcts[Title/Abstract])) OR (Heart Attack[Title/Abstract])) OR (Heart Attacks[Title/Abstract]))
3. 1 or 2
4. ("ST Elevation Myocardial Infarction"[Mesh])
5. (((ST Segment Elevation Myocardial Infarction[Title/Abstract]) OR (ST Elevated Myocardial Infarction[Title/Abstract])) OR (STEMI[Title/Abstract]))
6. 4 or 5
7. ("Non-ST Elevated Myocardial Infarction"[Mesh])
8. (((((((((Non ST Elevated Myocardial Infarction[Title/Abstract]) OR (NSTEMI[Title/Abstract])) OR (Non-ST-Elevation Myocardial Infarction[Title/Abstract])) OR (Infarction, Non-ST-Elevation Myocardial[Title/Abstract])) OR (Infarctions, Non-ST-Elevation Myocardial[Title/Abstract])) OR (Myocardial Infarction, Non-ST-Elevation[Title/Abstract])) OR (Myocardial Infarctions, Non-ST-Elevation[Title/Abstract])) OR (Non ST Elevation Myocardial Infarction[Title/Abstract])) OR (Non-ST-Elevation Myocardial Infarctions[Title/Abstract]))
9. 7 or 8
10. ("Acute Coronary Syndrome"[Mesh])
11. (((((Acute Coronary Syndromes[Title/Abstract]) OR (Coronary Syndrome, Acute[Title/Abstract])) OR (Coronary Syndromes, Acute[Title/Abstract])) OR (Syndrome, Acute Coronary[Title/Abstract])) OR (Syndromes, Acute Coronary[Title/Abstract]))
12. 10 or 11
13. 3 or 6 or 9 or 12
14. ((Xience) OR (everolimus-eluting stent))

15. (("Drug-Eluting Stents"[Mesh]) OR ((((((((((((Drug Eluting Stents[Title/Abstract]) OR (Stents, Drug-Eluting[Title/Abstract])) OR (Stents, Drug Eluting[Title/Abstract])) OR (Drug-Eluting Stent[Title/Abstract])) OR (Drug Eluting Stent[Title/Abstract])) OR (Stent, Drug-Eluting[Title/Abstract])) OR (Drug-Coated Stents[Title/Abstract])) OR (Drug Coated Stents[Title/Abstract])) OR (Stents, Drug-Coated[Title/Abstract])) OR (Stents, Drug Coated[Title/Abstract])) OR (Drug-Coated Stent[Title/Abstract])) OR (Drug Coated Stent[Title/Abstract])) OR (Stent, Drug-Coated[Title/Abstract])))

16. 14 or 15

17. (((((bioabsorbable scaffold) OR (bioresorbable scaffold)) OR (bioabsorbable stent)) OR (bioresorbable stent)) OR (BVS)) OR (BRS)

18. ((randomized controlled trial[Publication Type] OR randomized[Title/Abstract] OR placebo[Title/Abstract]))

19. 13 and 16 and 17 and 18

Excluded records after full-text screening (1-18) :

Publications on same trial (n=1)

1. Rai H, Alfonso F, Maeng M, et al. Optical coherence tomography tissue coverage and characterization at six months after implantation of bioresorbable scaffolds versus conventional everolimus eluting stents in the ISAR-Absorb MI trial. Int J Cardiovasc Imaging. 2021;37(10):2815-2826. doi:10.1007/s10554-021-02251-x.

Not Myocardial Infarction (n=4)

2. Schukraft, S., Arroyo, D., Togni, M., et al. Five-year angiographic, OCT and clinical outcomes of a randomized comparison of everolimus and biolimus-eluting coronary stents with everolimus-eluting bioresorbable vascular scaffolds. Catheterization and Cardiovascular Interventions. 2022;99(3), 523-532. doi:10.1002/ccd.29837.
3. Arroyo D, Gendre G, Schukraft S, et al. Comparison of everolimus- and biolimus-eluting coronary stents with everolimus-eluting bioresorbable vascular scaffolds: Two-year clinical outcomes of the EVERBIO II trial. Int J Cardiol. 2017;243:121-125. doi:10.1016/j.ijcard.2017.05.053.
4. Kereiakes DJ, Ellis SG, Metzger C, et al. 3-Year Clinical Outcomes With Everolimus-Eluting Bioresorbable Coronary Scaffolds: The ABSORB III Trial. J Am Coll Cardiol. 2017;70(23):2852-2862. doi:10.1016/j.jacc.2017.10.010.

5. Smits PC, Chang CC, Chevalier B, et al. Bioresorbable vascular scaffold versus metallic drug-eluting stent in patients at high risk of restenosis: the COMPARE-ABSORB randomised clinical trial. EuroIntervention. 2020;16(8):645-653. doi:10.4244/EIJ-D-19-01079.

Not targeted outcome (n=4)

6. Yamaji, K., Raber, L., Brugaletta, S., et al. Effect of post-dilatation on device expansion and arterial healing following Everolimus-eluting bioresorbable stent (Absorb BVS) versus durable polymer everolimus-eluting metallic stent (EES) implantation in STEMI patients: substudy of ABSORB STEMI TROFI II. Journal of the American College of Cardiology.2016;68:18 (B162) Supplement 1.https://www.cochranelibrary.com/central/doi/10.1002/central/CN-01475110/full.
7. Yamaji, K., Brugaletta, S., Sabaté, M., et al. Effect of Post-Dilatation Following Primary PCI With Everolimus-Eluting Bioresorbable Scaffold Versus Everolimus-Eluting Metallic Stent Implantation: An Angiographic and Optical Coherence Tomography TROFI II Substudy. JACC Cardiovasc Interv.2017; 10(18), 1867-1877. doi:10.1016/j.jcin.2017.07.035.
8. Brugaletta, S., Cequier, A., Alfonso, F.,et al. MAGnesium-based bioresorbable scaffold and vasomotor function in patients with acute ST segment elevation myocardial infarction: the MAGSTEMI trial: rationale and design. Catheterization and Cardiovascular Interventions.2019;93(1), 64-70. doi:10.1002/ccd.27825.
9. Räber L, Onuma Y, Brugaletta S, et al. Arterial healing following primary PCI using the Absorb everolimus-eluting bioresorbable vascular scaffold (Absorb BVS) versus the durable polymer everolimus-eluting metallic stent (XIENCE) in patients with acute ST-elevation myocardial infarction: rationale and design of the randomised TROFI II study. EuroIntervention. 2016;12(4):482-489. doi:10.4244/EIJY15M08_03.

Not targeted intervention or control (n=1)

10. Sabaté M, Brugaletta S, Cequier A, et al. Clinical outcomes in patients with ST-segment elevation myocardial infarction treated with everolimus-eluting stents versus bare-metal stents (EXAMINATION): 5-year results of a randomised trial. Lancet. 2016;387(10016):357-366. doi:10.1016/S0140-6736(15)00548-6.

Not randomized controlled trial (n=2)

11. Albani, S., Pisano, F., Bernardi, A.,et al. First generation bioresorbable vascular scaffold vs next generation bioresorbable vascular scaffold: Single center, long-term follow-up experience. Giornale Italiano di Cardiologia.2021; 22(SUPPL 1), e32-e33.https://www-embase-com.ezproxy.lib.asia.edu.tw:2443/records?subaction=viewrecord&rid=1&page=1&id=L636443876.

12. Brugaletta S, Gori T, Low AF, et al. Absorb bioresorbable vascular scaffold versus everolimus-eluting metallic stent in ST-segment elevation myocardial infarction: 1-year results of a propensity score matching comparison: the BVS-EXAMINATION Study (bioresorbable vascular scaffold-a clinical evaluation of everolimus eluting coronary stents in the treatment of patients with ST-segment elevation myocardial infarction) [published correction appears in JACC Cardiovasc Interv. 2015 Mar;8(3):503]. JACC Cardiovasc Interv. 2015;8(1 Pt B):189-197. doi:10.1016/j.jcin.2014.10.005.

Abstracts with no full text(n=6)

13. Wiebe, J., Schneider, S., Cassese, S., et al.TCT-11 Everolimus-Eluting Bioresorbable Scaffolds Versus Drug-Eluting Stents in Patients With Acute Myocardial Infarction: 2-Year Results of the Randomized ISAR-Absorb MI Trial. Journal of the American College of Cardiology.2019; 74(13), B11. doi:10.1016/j.jacc.2019.08.034.
14. Rai H, Alfonso F, Maeng M, et al.51 Neointimal tissue coverage and characterization assessment using optical coherence tomography surveillance six months after implantation of bioresorbable scaffolds versus conventional everolimus eluting stents in ISAR-absorb MI trial.Heart 2020;106:A33. doi:10.1136/heartjnl-2020-ICS.51.
15. Rai, H., Alfonso, F., Maeng, M.,et al.Morphometric and qualitative differences in neointimal tissue six months after implantation of bioresorbable scaffolds versus conventional everolimus eluting stents in ISAR-Absorb MI trial. European Heart Journal.2019;40, 3502. doi:10.1093/eurheartj/ehz746.0574.
16. Rai, H., Alfonso, F., Maeng, M.,et al.Optical coherence tomography tissue coverage and characterization at six months after implantation of bioresorbable scaffolds versus conventional everolimus eluting stents in the ISAR-Absorb MI trial. European Heart Journal.2018;39, 526-527. doi:10.1093/eurheartj/ehy565.P2632.
17. Rai, H., Alfonso, F., Maeng, M.,et al.Optical coherence tomography surveillance of tissue coverage at six months after stenting for acute myocardial infarction with bioabsorbable vascular scaffolds in the ISAR-Absorb MI trial. Journal of the American College of Cardiology.2017; 70(18), B168-B169. doi:10.1016/j.jacc.2017.09.509.
18. Rai, H., Alfonso, F., Maeng, M., et al.TCT-180 Neointimal Tissue Coverage and Characterization Assessment Using Optical Coherence Tomography Surveillance 6 Months After Implantation of Bioresorbable Scaffolds Versus Conventional Everolimus-Eluting Stents in ISAR-Absorb MI Trial. Journal of the American College of Cardiology.2019;74(13), B179. doi:10.1016/j.jacc.2019.08.237.

REFERENCES

1. Higgins JP, Altman DG, Gøtzsche PC, Jüni P, Moher D, Oxman AD, et al. The Cochrane Collaboration's tool for assessing risk of bias in randomised trials. BMJ. (2011);343:d5928. doi: 10.1136/bmj.d5928
2. de la Torre Hernandez JM, Garcia Camarero T, Lee DH, Sainz Laso F, Veiga Fernandez G, Pino T, et al. Procedural resources utilization and clinical outcomes with bioresorbable everolimus-eluting scaffolds and Pt-Cr everolimus-eluting stent with resorbable abluminal polymer in clinical practice. A randomized trial. Catheter Cardiovasc Interv. (2017);90:E25-E30. doi: 10.1002/ccd.26843
3. Katagiri Y, Onuma Y, Asano T, Iñiguez A, Jensen LO, Cequier À, et al. Three-year follow-up of the randomised comparison between an everolimus-eluting bioresorbable scaffold and a durable polymer everolimus-eluting metallic stent in patients with ST-segment elevation myocardial infarction (TROFI II trial). EuroIntervention. (2018);14:e1224-e1226. doi: 10.4244/EIJ-D-18-00839
4. Wiebe J, Byrne RA, Alfonso F, Maeng M, Bradaric C, Kretov E, et al. Clinical outcomes of everolimus-eluting bioresorbable scaffolds or everolimus-eluting stents in patients with acute myocardial infarction: two-year results of the randomised ISAR-Absorb MI trial. EuroIntervention. (2022);17:1348-1351. doi: 10.4244/EIJ-D-21-00653
5. Sabaté M, Alfonso F, Cequier A, Romaní S, Bordes P, Serra A, et al. Magnesium-Based Resorbable Scaffold Versus Permanent Metallic Sirolimus-Eluting Stent in Patients With ST-Segment Elevation Myocardial Infarction: The MAGSTEMI Randomized Clinical Trial. Circulation. (2019);140:1904-1916. doi: 10.1161/CIRCULATIONAHA.119.043467
